# Phyllosphere bacterial community and metabolomic analysis revealed the mechanism of Cd tolerance in the bryophyte *Tortella tortuosa* (Hedw.) Limpr

**DOI:** 10.3389/fpls.2024.1466659

**Published:** 2024-11-28

**Authors:** Yongqi Zhu, Qiuge Li, Lanlan Feng, Yijie Dong, Yuanpei Zhang, Nigati Nurmaimaiti, Reyim Mamut

**Affiliations:** Key Laboratory of Biological Resources and Genetic Engineering of Xinjiang, College of Life Science and Technology, Xinjiang University, Urumqi, China

**Keywords:** antioxidant enzymes, bacterial diversity, cadmium stress, differentially abundant metabolites, lichen

## Abstract

**Introduction:**

Phytoremediation is a safe and green technology for the remediation of heavy metal pollution, a global environmental problem. Bryophytes are well known for their special physiological properties, but there is little research on the use of bryophytes for phytoremediation.

**Methods:**

In this indoor experiment, the impacts of 40 days of Cd pollution (1 (T1), 5 (T2), 10 (T3) mg·L^-1^) on Cd absorption, growth and physiological characteristics, and phyllosphere bacterial diversity of *Tortella tortuosa* were explored.

**Results:**

The results showed that the maximum Cd absorption capacity of *T. tortuosa* was 5.0135 mg·kg^-1^. The contents of leaf chlorophyll a (*Chl a*) and chlorophyll b (*Chl b*) in *T. tortuosa* decreased (*p* < 0.05) with the increase of Cd concentration. Especially, the Chl a and Chl b contents of the T3 treatment reduced by 88% and 91%, respectively compared with those of the CK (Cd: 0 mg·L^-1^). The catalase (CAT) and peroxidase (POD) activities of the T3 treatment reduced by 55% and 85%, respectively (*p* < 0.05), and the malondialdehyde (MDA) content increased by 167%, compared with those of the CK. Under Cd exposure, Cyanobacteria (63.49%) and Proteobacteria (26.62%) were the dominant bacterial phyla. The highly abundant phyllosphere bacteria were negatively correlated with the Cd concentration, antioxidant enzyme activity, and chlorophyll content in *T. tortuosa*, and positively correlated with the relative abundances of Neomycin and N-Acetyl-L-Glutamic acid.

**Discussion:**

Although the severe Cd pollution could affect the physiological and metabolic characteristics of *T. tortuosa*, *T. tortuosa* had a strong absorption capacity for Cd. Therefore, it could be used for phytoremediation of heavy metal pollution. This study will provide a reference for the remediation of soil heavy metal pollution.

## 1 Introduction

Heavy metals can not only cause environment pollution, but also threaten human health (Mateos et al., 2018; Wang et al., 2024). Cadmium (Cd) is the main pollutant affecting soil quality in China (Zhang and Reynolds, 2019; Zhang et al., 2024). Previous studies have shown that Cd can reduce plant nutrient absorption by inhibiting plant root growth and even causing root rot, and disrupt plants’ internal homeostasis by causing metabolic disorders and damaging the enzyme systems involved in the metabolism (Feng et al., 2021; Yang et al., 2022; Ghorbani et al., 2024). Besides, Cd can also greatly impact the physiological and biochemical characteristics of bryophytes. Lightly Cd pollution can reduce the chlorophyll content and antioxidant enzyme activities of bryophytes, and severe Cd pollution can obviously inhibit bryophyte growth.

Bryophytes have strong pollutant adsorption capacity (extracellular and intracellular absorption) owing to their large leaves and abundant cation exchange sites (Boquete et al., 2021). Although bryophytes have a certain tolerance to heavy metals, high concentrations of heavy metals (i.e., Cd > 1 μg·L^-1^ and Zn > 18 μg·L^-1^) can still damage the photosynthetic system and intracellular ultrastructure of bryophytes, cause lipid peroxidation of cell membranes, and greatly impact their physiological and biochemical activities (Maresca et al., 2023). For example, Rau et al. (2007) and Koz and Cevik (2014) reported that under Cd stress, chlorophyll in bryophyte cells was degraded, and glutathione chelated Cd in cytoplasmic transport. Esposito et al. (2018) and Printarakul et al. (2022) reported that Cd stress induced membrane lipid peroxidation in bryophytes by disrupting the metabolic balance of free radicals, resulting in oxidative stress. Maresca et al. (2022) and Sheng et al. (2022) reported that with the increase of Cd concentration, the contents of reactive oxygen species, free radicals, malondialdehyde, and the activity of peroxidase in bryophytes gradually increased, but the catalase activity decreased. Peroxidase plays an important role in scavenging excessive reactive oxygen species (ROS) produced under heavy metal stress (Zhan et al., 2014; Renzaglia et al., 2024). Malondialdehyde content and catalase activity respond quickly to the variations of heavy metal concentration, and have been used as biomarkers to monitor heavy metal pollution (Sun et al., 2009). Bryophytes resist heavy metal stresses mainly by accumulating toxic elements in cell walls and vacuoles, (2) changing the antioxidant system, and (3) secreting phytochelatins (De Agostini et al., 2022).

However, the impacts of heavy metal pollution are not only manifested in the changes in the physiological and metabolic characteristics of bryophytes, but also in the diversity and structure of microbial communities. Microbial communities either play an active role in the detoxification or adapt to adverse environments (Cernava et al., 2018). Chen et al. (2023) showed that the dominant families of bryophytes in a manganese mining area were Bryaceae and Pottiaceae, the dominant bacterial phyla in the soil under moss cover were Actinobacteriota, Proteobacteria, Chloroflexi, Acidobacteriota, and Gemmatimonadota, and the soil bacterial community structure was significantly affected by heavy metals. In addition, there were significant differences in the diversity and structure of rhizobial bacterial community in bryophytes under different degrees of heavy metal pollution, and most of rhizobial bacteria had strong tolerance (Ma et al, 2024). However, the correlation between the changes of bryophyte phyllosphere microbial community and the changes in bryophyte metabolic and physiological characteristics and the role played by bryophyte phyllosphere microorganisms in Cd pollution remediation are still unclear.

Bryophytes respond quickly to variations in heavy metal and organic pollutant contents, and have been used as excellent bio-monitors for monitoring environmental pollution (Konno et al., 2010; Boquete et al., 2021, Boquete et al., 2022). However, the physiological responses to Cd and Cd tolerance mechanisms of bryophytes have not been fully elucidated. Therefore, in this indoor experiment, the physiological and metabolic responses of *T. tortuosa* in the Tianshan Mountains in Xinjiang, China to simulated Cd pollution (1, 5, and 10 mg Cd L^-1^) were explored by analyzing the diversity of phyllosphere bacterial communities and differentially abundant metabolites (DAMs). This study hypothesized that *T. tortuosa* (1) *T. tortuosa* might resist exogenous Cd stress by adjusting its physiological activities and phyllosphere bacterial diversity, and (2) *T. tortuosa* might have great potential for phytoremediation of Cd pollution (Tian et al., 2022). The objectives were to explore (1) the Cd absorption capacity of *T. tortuosa* under the conditions of different concentrations of Cd, (2) the impact of different concentrations of Cd on the physiological characteristics of *T. tortuosa*, and (3) the changes in phyllosphere bacterial diversity and metabolome of *T. tortuosa* under Cd stress. This study will be of great significance for the development and utilization of bryophytes, and provide a scientific basis for the remediation of heavy metal pollution by using *T. tortuosa*.

## 2 Materials and methods

### 2.1 Collection and cultivation of *T. tortuosa*

*Tortella tortuosa* samples were collected in the Tianshan Mountains in Urumqi, China (43°13′51” N, 86°30′55” E; 1450 m a.s.l.). The annual average maximum temperature in the sampling site was 38°C, the minimum temperature was -20°C, the average temperature in winter was -15°C, and the average temperature in summer was 23°C. The annual average precipitation was 2084 mm, the annual average evaporation was 2616.9 mm, the annual average frost-free period was 179 days, and the annual average sunshine duration was 2813.5 hours.

The collected *T. tortuosa* samples were washed with water and placed in a hydroponic device. Knudoson C nutrient solution consisting of 1000 mL of distilled water, 1000 mg of Ca(NO_3_)_2_·4H_2_O, 250 mg of KH_2_PO_4_, 500 mg of (NH_4_)SO_4_, 25 mg of FeSO_4_·7H_2_O, 250 mg of MgSO_4_·7H_2_O, and 7.5 mg of MnSO_4_·4H_2_O was prepared. Then, 60 mL of Knudoson C nutrient solution was added into the hydroponic device, followed by culturing in an incubator (light intensity: 2000 lux; light time: 16 h; humidity: 75%; temperature: 20°C). The hydroponic solution was not replaced until the end of the culturing.

### 2.2 Experimental design

Pretest results showed that when the Cd concentration in the hydroponic solution reached 10 mg· L^-1^, *T. tortuosa* plants showed severe wilting. Therefore, three Cd pollution treatments ((1 (T1), 5 (T2), 10 (T3) mg Cd L^-1^)) and a control treatment (CK, 0 mg Cd L^-1^) were designed in the study. Each treatment had three replicates/pots (12 pots in total). The diameter of the pots was 15 cm, and the height was 10 cm. Plants (8.0 g) were placed in a transparent box, and 60 mL of Cd solution (0, 1, 5, and 10 mg Cd L^-1^) prepared with CdCl_2_·2.5H_2_O and ultrapure water were sprayed on plant surface every three days (12 times in total). The growth, physiological, and biochemical parameters of *T. tortuosa* were measured and recorded regularly. To suppress algae growth, the medium was diluted 10 times to reduce the concentration of phosphorus and nitrogen by 66% (Rau et al., 2007). After 50 days, a portion of fresh samples was used to determine fresh weight, dry weight, enzyme activity, and Cd concentration, and the remaining samples were stored at -80°C for analysis of phyllosphere bacterial diversity and metabolomics.

### 2.3 Measurement methods

#### 2.3.1 Measurement of Cd concentration and physiological parameters in *T. tortuosa*

One plant sample was taken from each of the three replicates of each treatment to measure the Cd concentration and physiological parameters, and the averages were calculated. Plant samples were dried to constant weight, cut into pieces, and pulverized in a cryo-grinder. About 0.5 g of pulverized samples were transferred to a microwave digestion tank, and 5 mL of nitric acid were slowly added to fully rinse the side wall of the digestion tank, so that the samples were completely infiltrated with nitric acid solution. After one night, microwave digestion was started. After cooling, the digestion tank was taken out to gradually release nitrogen dioxide. The digestion solution was transferred to a 50 mL measuring cylinder. The digestion tank was washed multiple times with a small amount of water, and the water was also transferred to the measuring cylinder. A sample blank solution was prepared simultaneously by the same method. Finally, the Cd concentration was determined by a spectrophotometer (Hitachi Z2000, Tokyo, Japan).

Acetone (80%) was used to extract chlorophyll, followed by colorimetry at 663 nm and 646 nm. The Lichtenthaler equation was used to calculate the contents of *Chl a* and *Chl b* (Shakya et al., 2008). The activity of catalase (CAT), superoxide dismutase (SOD), peroxidase (POD), and the content of malondialdehyde (MDA) were determined by the methods of Paoletti et al. (1986); Cakmak and Marschner (1992), and Bharwana et al. (2014).

#### 2.3.2 Analysis of metabonome

Two plant samples were taken from each of the three replicates of CK and T3 treatments to analyze the metabonome. Plant samples were rinsed, cut into pieces, and frozen in liquid nitrogen. Samples (100 mg) were ground with liquid nitrogen, and then 500 μL of 80% methanol aqueous solution was added, followed by vortexing and 5-min ice bath. After centrifuging for 20 min (4°C, 15000 g), the supernatant was taken, and water was added to give the solution a methanol content of 53%. After centrifuging for 20 min (4°C, 15000 g), the supernatant was taken for subsequent analysis. Quality control (QC) samples were equal-volume samples prepared by mixing the samples, and 53% methanol aqueous solution was used to prepare the sample blank solution (Chen et al., 2019).

Chromatography was carried out using a chromatograph (Vanquish UHPLC, Thermo Fisher) and a chromatographic column (Hypesil Goldcolumn, Thermo Fisher). The column temperature was 40°C, and the flow rate was 0.2 mL/min. In positive mode, the mobile phase A was 0.1% formic acid, and the mobile phase B was methanol. In negative mode, the mobile phase A was 5 mmol/L ammonium acetate (pH: 9.0), and the mobile phase B was methanol. Mass spectrometry was carried out using a mass spectrometer (Q Exactive™HF, Thermo Fisher). The spray voltage was 3.5 kV, the sheath gas flow rate was 35 psi, the aux gas flow rate was 10 L/min, the capillary temperature was 320°C, the S-lens RF level was 60, the aux gas heater temperature was 350°C, and the scan range was 100-1500 m/z. In this study, only the samples from the CK and T3 treatments were comparatively analyzed. The DAMs detected in CK *vs* T3 are shown in **Supplementary Figure S2**.

#### 2.3.3 Analysis of phyllosphere bacteria

One plant sample was taken from each of the three replicates of CK and T3 treatments to analyze the phyllosphere bacterial diversity. To remove the dusts and microbes on the leaf surface, leaf samples were mixed with 400 µL of solution composed of ddH_2_O and Tween 20 (1: 1,000) in a centrifuge tube, followed by shaking for 15 min at 200 rpm. After removing the solution by a pipette, 400 µL of 75% ethanol was added, followed by 5-min shaking. Then, the solution was removed again, and 800 µL of sterile water was added, followed by vortexing for five times (30 s for each). Finally, the solution was removed using a pipette.

Nine DNA samples from each plant were mixed into one sample. Then, the V3–V4 region of the 16S rRNA gene of phyllosphere bacteria was sequenced by Maxim Bioengineering Co., Ltd., China using primers 341F (5′-CCTACGGGGG CWGCAG-3′) and 805R (5′-GACTACHVGGGT ATCTAATCC-3′) on the Illumina Miseq sequencing platform. PRINSEQ software was used to crop and filter each sample sequence to obtain the optimized reads. After removing the redundant sequences and merging, the sequences without duplicates were removed, and the duplicated sequences were clustered by operational taxonomic units (OTUs) according to the similarity level of 97%. Chimeras were removed during clustering. Then, the OTU representative sequences were obtained, and the OTU abundance table was generated. Finally, the Ribosomal Database Project (RDP) database was used to align the representative sequences to obtain taxonomic information of each OTU, and the composition of phyllosphere bacterial community for each sample of the T3 treatment was determined at the domain, phylum, class, order, family, and genus levels (Kiran and Prasad, 2019; Romanowski et al., 2023).

### 2.4 Data analysis

Statistical analysis, one-way ANOVA analysis, and Duncan’s multiple comparison test were performed on the Cd concentration, antioxidant enzyme activity, and chlorophyll content of *T. tortuosa* (*p* < 0.05) using SPSS software version 25.0 (SPSS Inc., Chicago, USA) and Origin software version 8.0 (Origin Lab, Massachusetts, USA). Plotting was completed using Origin software version 8.0 (Origin Lab, Massachusetts, USA). Charts were drawn using Adobe Illustrator CS6 (Adobe, USA). Correlation analysis was conducted using the R software version 4.2.1, and networks were drawn with the Gephi software version 0.9.5.

## 3 Results

### 3.1 Cadmium concentration in *T. tortuosa* plants

**Figure 1:**
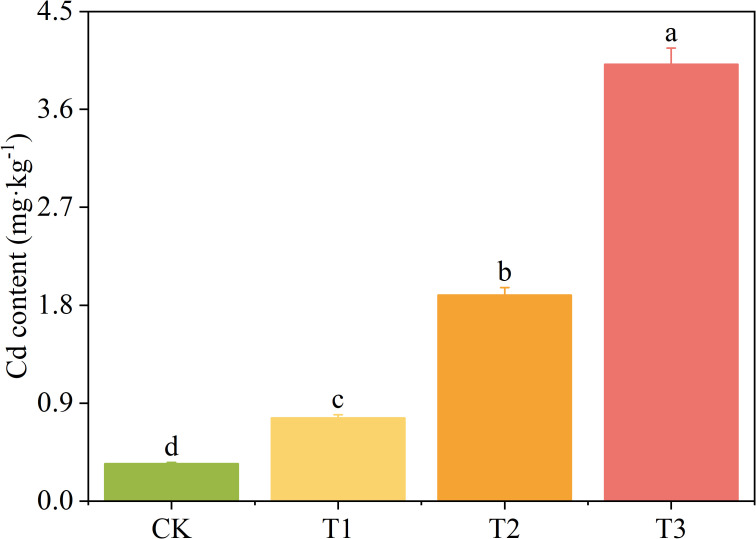
The Cd concentration in *Tortella tortuosa*. Values are mean ± SE (n = 5). Different lowercase letters indicate significant difference between groups at *p* < 0.05. T1, 1 mg·L^-1^ of Cd was applied; T2, 5 mg·L^-1^ of Cd was applied; T3, 10 mg·L^-1^ of Cd was applied. The same below.

The Cd concentration in *T. tortuosa* plants increased with the increase of exogenous Cd concentration (**Figure 1**). The plant Cd concentration of the T1, T2, and T3 treatments increased by 122.5%, 450.7%, and 968.1%, respectively (*p* < 0.05) compared with that of the CK.

### 3.2 Changes in leaf *Chl a* and *Chl b* contents of *T. tortuosa*

**Figure 2:**
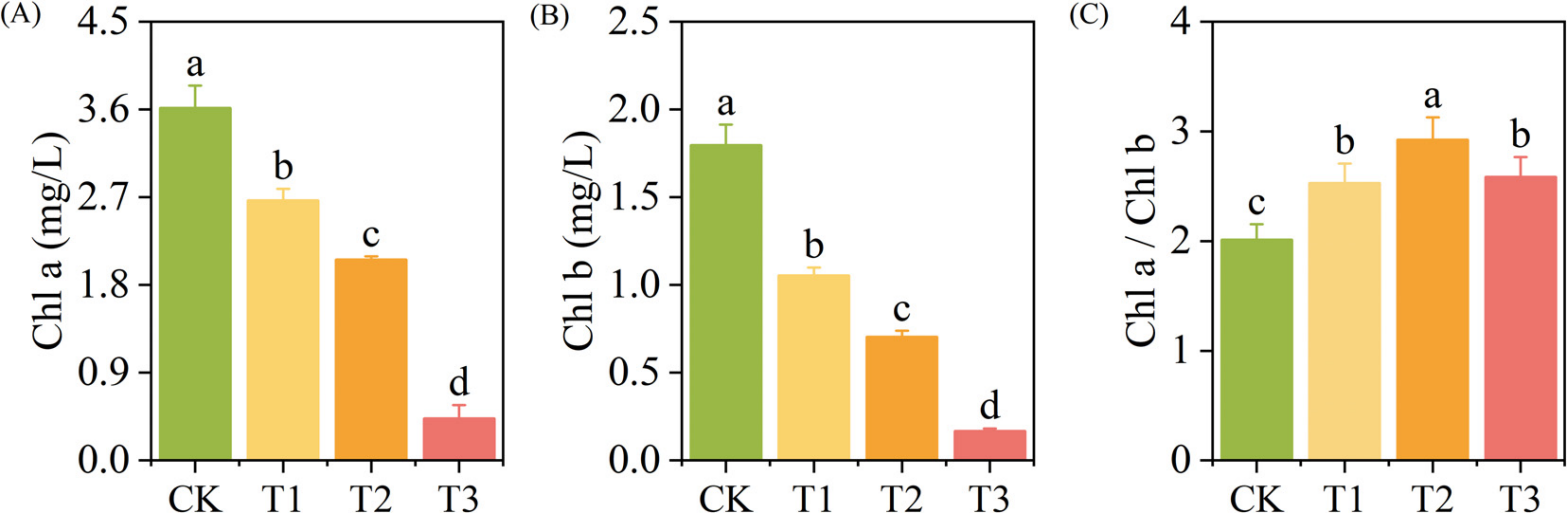
The content of chlorophyll a **(A)**, chlorophyll b **(B)**, and Chl a/Chl b **(C)** of *T. tortuosa*.

The contents of *Chl a* and *Chl b* decreased with the increase of exogenous Cd concentration (*p* < 0.05). The *Chl a* content of the T1, T2, and T3 treatments reduced by 23%, 43%, and 88%, respectively (*p* < 0.05) (**Figure 2A**), the *Chl b* content reduced by 41%, 61%, and 91%, respectively (*p* < 0.05) (**Figure 2B**), and the *Chl a*/*Chl b* increased by 25.69%, 45.26%, and 28.35%, respectively (*p* < 0.05) (**Figure 2C**), compared with those of the CK.

### 3.3 Changes in antioxidant enzyme activities in *T. tortuosa* leaves

**Figure 3:**
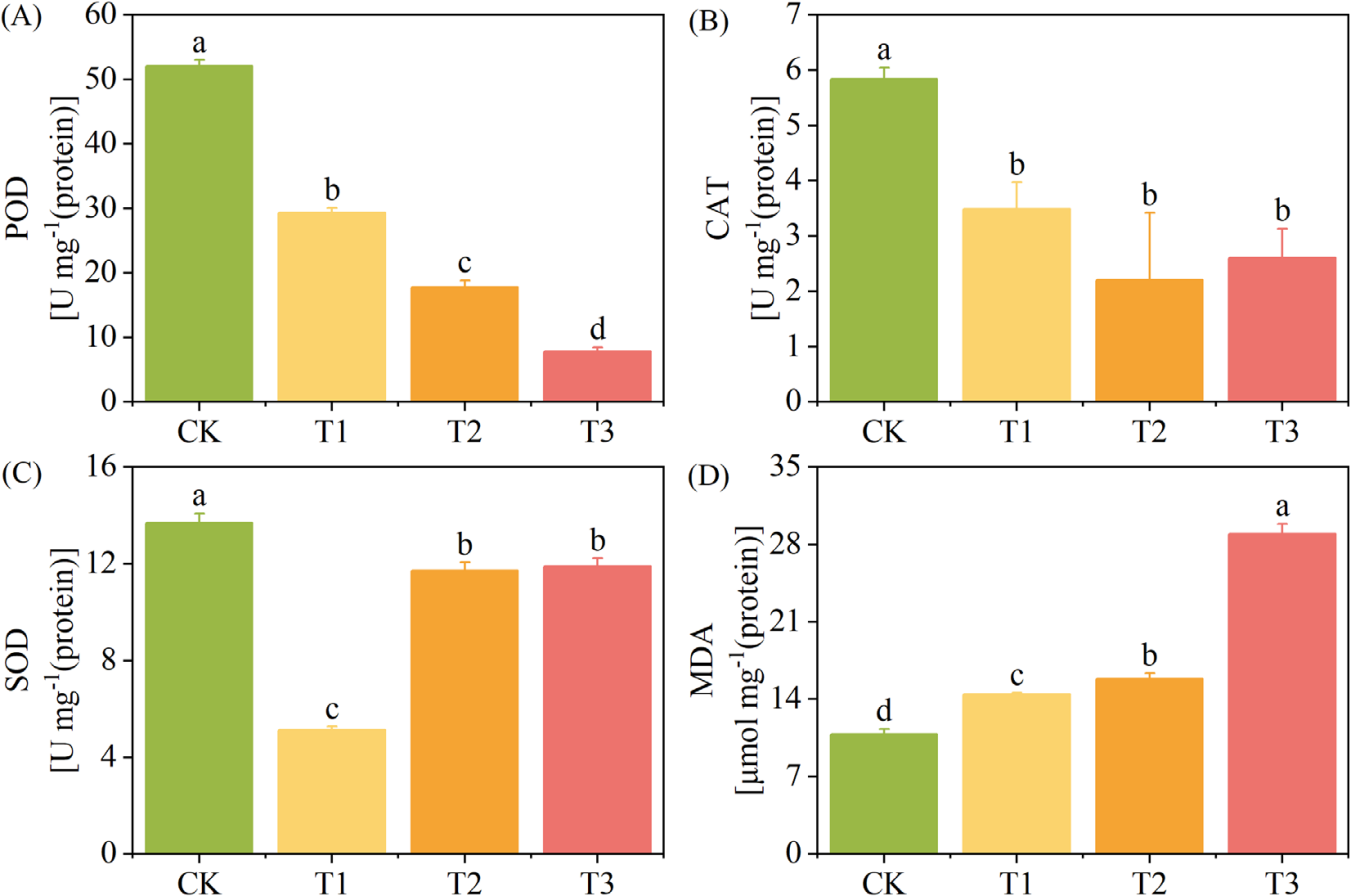
Antioxidant enzyme activity and malondialdehyde (MDA) content. of *T. tortuosa*. **(A)** Peroxidase (POD) activity; **(B)** Catalase (CAT) activity; **(C)** Superoxide dismutase (SOD) activity; **(D)** MDA content.

The activities of CAT and SOD decreased and then increased with the increase of exogenous Cd concentration (**Figure 3**). The POD activity of the T1, T2, and T3 treatments decreased by 44%, 66%, and 85%, respectively (*p* < 0.05) (**Figure 3A**), the CAT activity decreased by 40%, 62%, and 55% (*p* < 0.05), respectively (**Figure 3B**), and the SOD activity decreased by 63%, 14%, and 13%, respectively (*p* < 0.05) (**Figure 3C**), compared with those of the CK. The POD activity gradually decreased with the increase of exogenous Cd concentration, while the MDA content showed an opposite change trend. The MDA content of the T1, T2, and T3 treatments increased by 33%, 46%, and 167%, respectively compared with that of the CK (**Figure 3D**).

### 3.4 Changes in phyllosphere bacterial communities under Cd exposure

**Figure 4:**
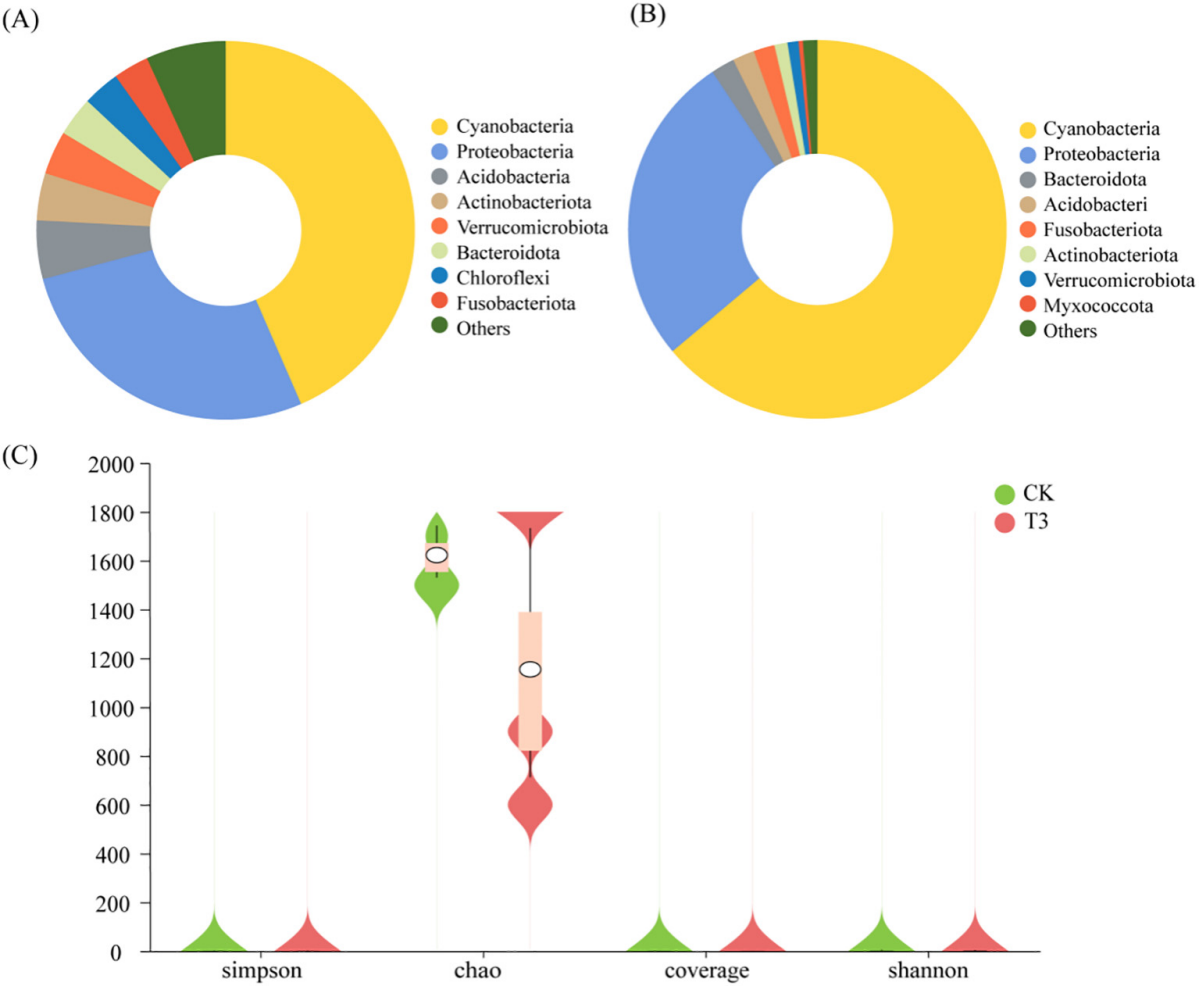
Analysis results of phyllosphere bacterial diversity of *T. tortuosa*. **(A)** Phyllosphere bacterial diversity of the CK treatment (species level); **(B)** Phyllosphere bacterial diversity of the T3 treatment (species level); **(C)** Phyllospheric bacterial α-diversity.

Cyanobacteria (relative abundance: 43.48%), Proteobacteria (27.38%), Acidobacteriota (5.00%), and Actinobacteriota (4.04%) were the dominant bacterial families in the CK (**Figure 4A**). Cyanobacteria (63.87%) and Proteobacteria (26.78%) were the dominant bacterial families in the T3 treatment (**Figure 4B**). The relative abundance of Cyanobacteria of the T3 treatment increased by 20.39%, and the relative abundances of Proteobacteria and Acidobacteriota decreased by 0.6% and 3.12%, respectively, compared with those of the CK. The analysis of α-diversity showed that there was a significant difference in Chao1 index between CK and T3 treatments, and the Chao1 index of the T3 treatment reduced by 28.87% compared with that of the CK (*p* < 0.05) (**Figure 4C**). The analysis of β-diversity found that there was difference in the bacterial composition between CK and T3 treatments (**Supplementary Figure S1**).

### 3.5 Comparative analysis of differentially abundant metabolites in *T. tortuosa* of different treatments

**Figure 5:**
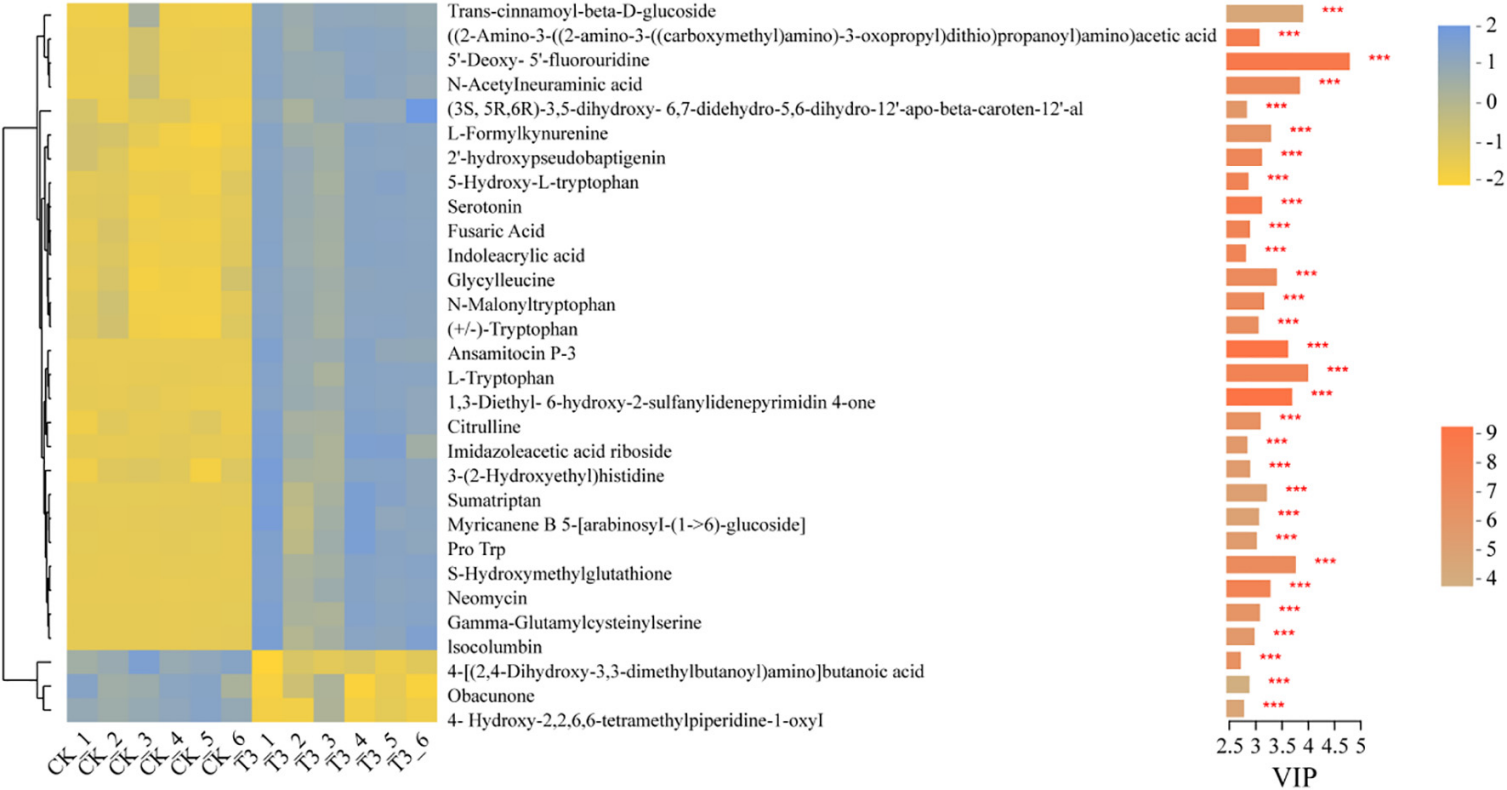
Analysis of differentially abundant metabolites (DAMs) based on the Kruskal-Wallis test. The bar color indicates the significant difference of metabolites between the two groups of samples, that is, the P value, the smaller the P, the larger the P value, the darker the color. * means P < 0.05, ** means P < 0.01, *** means P < 0.001.

A total of 30 DAMs (5’-Deoxy-5’-fluorouridine, N-Acetylneuraminic acid, Ansamitocin P-3, L-Tryptophan, S-Hydroxymethylglutathione, Neomycin, etc.) were detected (**Figure 5**), and their relative abundances were larger in the T3 treatment than in the CK (*p* < 0.001). Besides, these DAMs were mainly involved in the metabolic pathways such as Amino acids, Nucleotides, Carboxylic acids, and Steroid hormones (**Supplementary Figure S2**).

### 3.6 Correlation analysis of physiological characteristics, Cd concentration, differentially abundant metabolites, and phyllosphere bacterial communities

**Figure 6:**
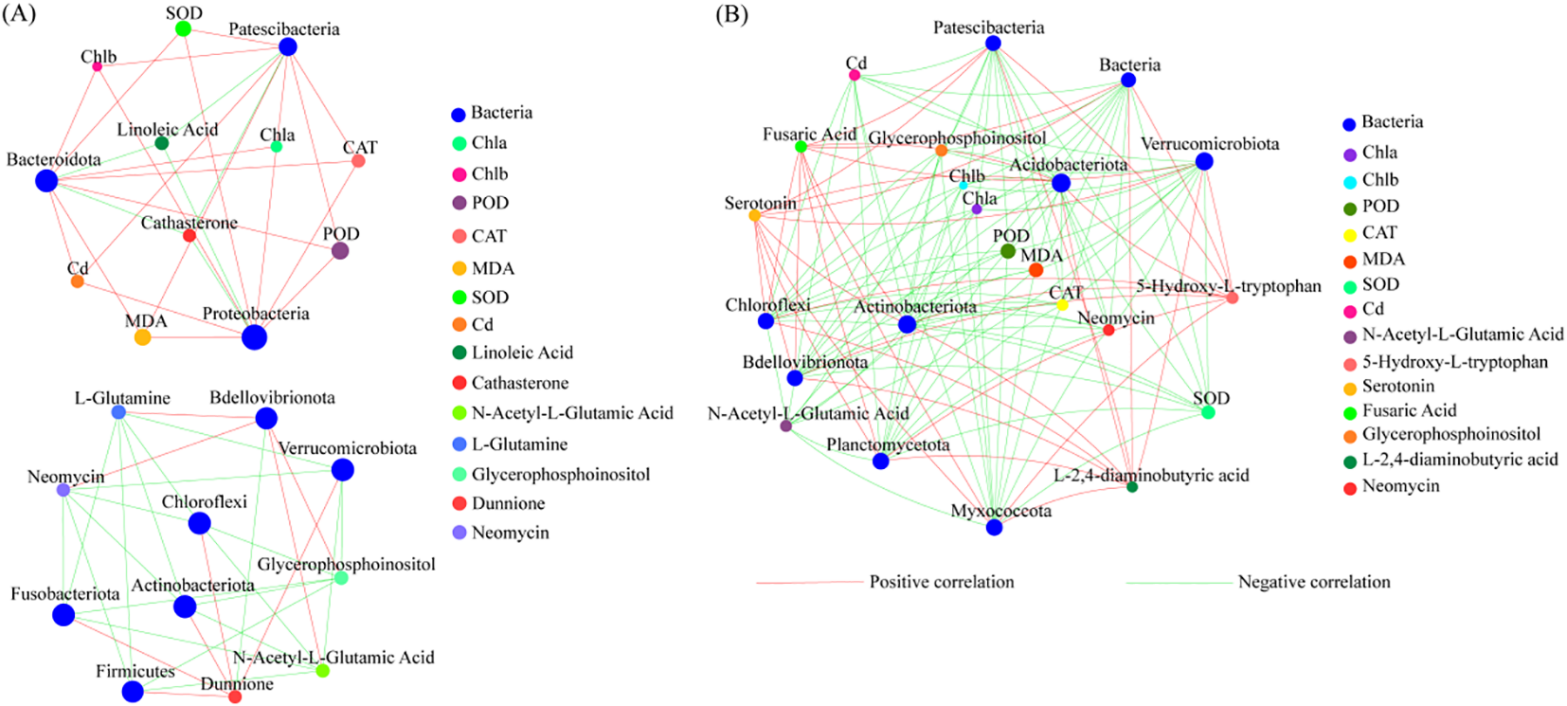
Correlation network between dominant bacterial species and physiological characteristics. **(A)** Correlation network of the CK treatment; **(B)** Correlation network of the T3 treatment. The network mainly reflects the species correlation under a certain environmental condition. The top 50 species by abundance at a taxonomic level were selected, and the correlation coefficients between bacteria, differentially abundant metabolites (DAMs), and physiological characteristics were presented. The size of the nodes in the figure indicates the abundance of species. Different colors indicate different factors. The red connecting lines indicate positive correlations, and the green connecting lines indicate negative correlations. The thicker the lines, the larger the correlation coefficients, and the higher the correlations. The more lines, the closer the correlations between bacterial abundance, DAMs, and plant physiological characteristics.

In the CK, the relative abundances of Patescibacteria, Bacteroidota, and Proteobacteria were correlated with the relative abundances of DAMs and the physiological characteristics of *T. tortuosa* leaves, i.e., the relative abundances of Patescibacteria and Bacteroidota were positively correlated with *Chl a* content, *Chl b* content, SOD activity, POD activity, and CAT activity (*p* < 0.05), and negatively correlated with the relative abundance of Linoleic acid (*p* < 0.05). The relative abundances of Bdellovibrionota, Verrucomicrobiota, Chloroflexi, Actinobacteriota, Fusobacteriota, and Firmicutes were negatively correlated with that of Dunnione (*p* < 0.05). The relative abundance of Bdellovibrionota was negatively correlated with that of L-Glytamine, Neomycin, Glycerophosphoinositol, and N-Acetyl-L-Glutamic acid (*p* < 0.05) (**Figure 6A**).

In the T3 treatment, the relative abundances of Acidobacteriota, Actinobacteriota, Verrucomicrobiota, Chloroflexi, Bdellovibrionota, Patescibacteria, and Planctomycetota were positively correlated with the relative abundances of Fusaric Acid, 5-Hydroxy-L-tryptophan, and Serotonin (*p* < 0.05). The relative abundances of Bdellovibrionota, Planctomycetota, Chloroflexi, Verrucomicrobiota, Acidobacteriota, Actinobacteriota, and Patescibacteria were positively correlated with the relative abundances of Neomycin and N-Acetyl-L-Glutamic Acid (*p* < 0.05). The relative abundances of Bdellovibrionota, Planctomycetota, Chloroflexi, Verrucomicrobiota, Acidobacteriota, Actinobacteriota, and Patescibacteria were negatively correlated with plant Cd concentration, SOD activity, POD activity, CAT activity, *Chl b* content, and *Chl a* content (*p* < 0.05) (**Figure 6B**).

The relative abundances of the DAMs N-Acetyl-L-Glutamic Acid, 5-Hydroxy-L-tryptophan, Serotonin, Fusaric Acid, L-Glutamine, Glycerophosphoinositol, L-Histidine, L-2,4-diaminobutyric acid, Dunnione, Linoleic Acid, Cathasterone, and Neomycin were negatively correlated with leaf Cd concentration and MDA content (*p* < 0.05), and positively correlated with SOD activity, POD activity, CAT activity, *Chl b* content, and *Chl a* content (*p* < 0.05) (**Table 1**). The relative abundances of the phyllosphere bacteria Actinobacteriota, Armatimonadota, Bdellovibrionota, Chloroflexi, Firmicutes, Planctomycetota, and Verrucomicrobiota were negatively correlated with leaf Cd concentration and MDA content (*p* < 0.05), and positively correlated with SOD activity, POD activity, CAT activity, *Chl b* content, and *Chl a* content (*p* < 0.05) (**Table 1**). The relative abundances of the DAMs N-Acetyl-L-Glutamic Acid, 5-Hydroxy-L-tryptophan, Serotonin, Fusaric Acid, L-Glutamine, Glycerophosphoinositol, L-Histidine, L-2,4-diaminobutyric acid, Dunnione, Linoleic Acid, Cathasterone, and Neomycin were negatively correlated with that of Cyanobacteria (*p* < 0.05), and positively correlated with the relative abundances of Actinobacteriota, Bdellovibrionota, and Verrucomicrobiota (*p* < 0.05) (**Table 2**).

**Table 1:** Pearson correlation analysis of physiological indexes, Cd content, phyllosphere bacterial community and differential metabolites of *T. tortuosa*.

Item	Cd	SOD	MDA	CAT	POD	*Chl a*	*Chl b*
N-Acetyl-L-Glutamic Acid	-0.897**	0.853**	-0.892**	0.906**	0.905**	0.905**	0.905**
5-Hydroxy-L-tryptophan	-0.977**	0.851**	-0.978**	0.966**	0.971**	0.972**	0.972**
Serotonin	-0.984**	0.856**	-0.984**	0.972**	0.978**	0.978**	0.978**
Fusaric Acid	-0.978**	0.849**	-0.979**	0.966**	0.972**	0.972**	0.972**
L-Glutamine	-0.917**	0.851**	-0.913**	0.921**	0.921**	0.921**	0.921**
Glycerophosphoinositol	-0.716**	0.748**	-0.707*	0.741**	0.734**	0.734**	0.733**
L-Histidine	-0.923**	0.781**	-0.925**	0.906**	0.913**	0.914**	0.914**
L-2,4-diaminobutyric acid	-0.904**	0.776**	-0.905**	0.890**	0.896**	0.897**	0.897**
Dunnione	-0.731**	0.488	-0.742**	0.682*	0.699*	0.700*	0.701*
Linoleic Acid	-0.910**	0.776**	-0.912**	0.895**	0.902**	0.902**	0.903**
Cathasterone	-0.885**	0.766**	-0.886**	0.873**	0.879**	0.879**	0.879**
Neomycin	-0.974**	0.893**	-0.971**	0.975**	0.977**	0.977**	0.977**
Acidobacteriota	-0.577*	0.428	-0.583*	0.55	0.56	0.561	0.561
Actinobacteriota	-0.948**	0.834**	-0.948**	0.939**	0.944**	0.944**	0.944**
Armatimonadota	-0.708**	0.641*	-0.706*	0.706*	0.708**	0.708**	0.708**
Bacteroidota	-0.472	0.295	-0.48	0.434	0.448	0.448	0.449
Bdellovibrionota	-0.974**	0.847**	-0.975**	0.962**	0.968**	0.968**	0.969**
Chloroflexi	-0.774**	0.805**	-0.765**	0.800**	0.793**	0.793**	0.792**
Cyanobacteria	0.758**	-0.610*	0.762**	-0.735**	-0.744**	-0.744**	-0.745**
Firmicutes	-0.628*	0.719**	-0.615*	0.667*	0.655*	0.654*	0.654*
Gemmatimonadota	-0.559	0.440	-0.563	0.540	0.547	0.548	0.548
Myxococcota	-0.416	0.288	-0.421	0.391	0.400	0.400	0.401
Patescibacteria	-0.030	-0.124	-0.041	-0.011	0.003	0.003	0.004
Planctomycetota	-0.807**	0.643*	-0.812**	0.782**	0.792**	0.792**	0.793**
Proteobacteria	-0.052	-0.148	-0.067	-0.001	0.017	0.018	0.019
Verrucomicrobiota	-0.985**	0.884**	-0.983**	0.980**	0.984**	0.984**	0.984**

* and ** represented significant correlation (*p* < 0.05) and extremely significant correlation (*p* < 0.01) between traits, respectively.

**Table 2:** Pearson correlation analysis between phyllosphere bacterial community and differential metabolites of *T. tortuosa*.

Item	N-Acetyl-L-Glutamic Acid	5-Hydroxy-L-tryptophan	Serotonin	Fusaric Acid	L-Glutamine	Glycerophosphoinositol	L-Histidine	L-2,4-diaminobutyric acid	Dunnione	Linoleic Acid	Cathasterone	Neomycin
Acidobacteriota	0.509	0.537	0.536	0.507	0.476	0.192	0.373	0.467	0.660*	0.48	0.606*	0.459
Actinobacteriota	0.839**	0.898**	0.915**	0.894**	0.860**	0.583*	0.808**	0.835**	0.646*	0.821**	0.835**	0.901**
Armatimonadota	0.493	0.633*	0.661*	0.666*	0.520	0.153	0.650*	0.502	0.562	0.693*	0.731**	0.632*
Bacteroidota	0.302	0.450	0.469	0.477	0.325	0.002	0.509	0.330	0.592*	0.542	0.692*	0.406
Bdellovibrionota	0.899**	0.972**	0.979**	0.973**	0.935**	0.787**	0.944**	0.918**	0.680*	0.905**	0.882**	0.983**
Chloroflexi	0.634*	0.684*	0.704*	0.691*	0.641*	0.382	0.592*	0.607*	0.429	0.639*	0.568	0.708*
Cyanobacteria	-0.559	-0.689*	-0.719**	-0.711**	-0.584*	-0.174	-0.677*	-0.568	-0.635*	-0.712**	-0.817**	-0.673*
Firmicutes	0.471	0.528	0.550	0.547	0.48	0.242	0.470	0.445	0.288	0.518	0.417	0.565
Gemmatimonadota	0.498	0.545	0.526	0.516	0.447	0.260	0.399	0.447	0.779**	0.524	0.614*	0.442
Myxococcota	0.368	0.388	0.376	0.353	0.314	0.088	0.219	0.313	0.638*	0.352	0.481	0.287
Patescibacteria	0.099	0.019	0.028	-0.020	0.089	-0.055	-0.117	0.086	0.019	-0.102	0.056	-0.008
Planctomycetota	0.792**	0.822**	0.836**	0.818**	0.845**	0.736**	0.807**	0.830**	0.451	0.716**	0.726**	0.856**
Proteobacteria	-0.063	0.060	0.081	0.089	-0.027	-0.283	0.177	-0.010	0.278	0.177	0.381	0.022
Verrucomicrobiota	0.874**	0.944**	0.958**	0.945**	0.898**	0.656*	0.876**	0.874**	0.675*	0.878**	0.865**	0.952**

* and ** represented significant correlation (*p* < 0.05) and extremely significant correlation (*p* < 0.01) between traits, respectively.

## 4 Discussion

### 4.1 Cd adsorption capacity and ecological restoration potential of *T. tortuosa*

The cell wall of bryophytes has a high cation exchange capacity, and plays an important role in the adsorption and binding of nutrients and heavy metals (Genty et al., 1989). This adsorption capacity of cell wall largely depends on the cell wall components (Maresca et al., 2023). A large number of organic groups on the cell wall of bryophytes absorb heavy metals in a passive and cation exchange manner, forming complexes. This prevents heavy metal ions from entering the cell protoplasm, and allows heavy metals to accumulate outside the cells, thus reducing the toxic effects (Tyler, 2010). In this study, the Cd concentration of *T. tortuosa* plants was as high as 5.01 mg·kg^-1^ when the exogenous Cd concentration was 10 mg· L^-1^ (**Figure 1**). This is due to the large leaf area of *T. tortuosa.* Koz and Cevik (2014) reported that *Eurhynchium striatum* had a higher Pb adsorption capacity than *Hypnum cupressiforme*, *Pleurozium schreberi*, *Eurhynchium striatum*, *Thuidium tamariscinum*, and *Homalothecium sericeum*, and the main reason for this difference was the difference in leaf area. Bryophytes can absorb heavy metal ions twice as much as seed plants, due to the larger surface area per unit of dry weight and the growth form (Schofield, 2001). However, when bryophytes absorb heavy metals up to a certain amount, their growth and development will be inhibited (Koz and Cevik, 2014). Shakya et al. (2008) found that under 10^-6^ M of CuCl_2_ treatment, the Cu content in *Thuidium sparsifolium*, *Thuidium delicatulum*, and *Ptychanthus striatus* (Lehm. & Lindenb.) was 18, 15, and 47 mg·kg^-1^, respectively, and Cu stress significantly suppressed the chlorophyll synthesis of the three species. Printarakul et al. (2022) also reported that the contents of Cu and Zn in *Scopelophila cataractae* were as high as 9903.5 and 271.8 mg·kg^-1^, respectively after two-week culture with Cu- and Zn-containing medium. Exogenous Cu addition induced increases in the contents of indoleacetic acid and isopentenyladenine in bryophytes, while these two were vital for the protonemal development. Besides, Printarakul et al. (2022) also found that exogenous heavy metal addition could inhibit the photosynthetic activity and chlorophyll synthesis of bryophytes, resulting in decreases in gametophyte survival and bryophyte biomass. Consistently, in this study, the *Chl a* and *Chl b* contents of *T. tortuosa* decreased with the increase of exogenous Cd concentration. That is, high Cd concentrations can interfere with *T. tortuosa*’s photosynthesis, growth, and development. It can be seen that bryophytes have strong heavy metal adsorption capacity. Thus, it can be used for phytoremediation of Cd pollution.

### 4.2 Responses of antioxidant enzyme activities in *T. tortuosa* to Cd stress

Plant Cd tolerance depends on the physicochemical tolerance, metal absorption capacity, and antioxidant capacity of plants (Mousavi et al., 2022). Many abiotic stresses, including heavy metal stress, cause damages to plant organelles by increasing ROS. Plant cells respond to the increase of ROS by changing the antioxidant system. The major enzymes in this system are CAT, SOD, and POD, which are vital for preventing plant organelles and tissues from oxidative damage (Esposito et al., 2018). Maresca et al. (2023) found that under Cd stress (3.3 μg Cd g^-1^), the CAT, SOD, and GST (glutathione S-transferase) activities of *Conocephalum conicum* L. increased with the increase of Cd concentration, and these enzymes jointly inhibited the production of ROS to resist the Cd stress. However, in this research, the CAT and POD activities in the leaves of *T. tortuosa* decreased with the increase of exogenous Cd concentration (**Figures 3A, B**). This difference is due to the fact that (1) Different bryophytes have different tolerance to Cd stress, and (2) the heavy metal and its concentration in this study are inconsistent with those of Maresca et al. (2023). The SOD can transform superoxide radicals (O_2_^−^) in the cytoplasm, chloroplasts, and mitochondria to H_2_O_2_ in Cd-stressed plants, and play a crucial role in suppressing hydroxyl radical formation (Wang et al., 2018). In this study, the SOD activity increased with the increase of exogenous Cd concentration. This indicates that *T. tortuosa* still has a certain ability to scavenge ROS under Cd stress (Wang et al., 2018). This may be due to the fact that (1) superoxide free radicals damage plant cell membranes, and free radicals could no longer be scavenged by increasing the activity of SOD alone. Then, the CAT activity is increased to scavenge H_2_O_2_ in the early stage of stress, to reduce the potential ROS damage. The SOD activity decreases when the peroxide production by the SOD-catalyzed disproportionation exceeds the removal capacity of POD and CAT (Wang et al., 2023). (2) Under low heavy metal concentration conditions, the antioxidant enzyme system of bryophytes can scavenge excess ROS and protect cells from damage through the synergistic action of multiple antioxidant enzymes. However, under high heavy metal concentration conditions, the homeostasis is disrupted, the oxidative stress is increased, and the accumulation rate of ROS exceeds the scavenging rate of the antioxidant enzyme system (Pan et al., 2020). The results of this study indicate that *T. tortuosa* mainly resists Cd stress by reducing the activities of POD and CAT. Plants often interfere with the production of ROS and MDA and scavenge ROS by increasing the antioxidant enzyme activities, and promote the synthesis of substances directly involved in the reaction with ROS (Yang, 2022). In this study, the content of MDA increased significantly with the increase of exogenous Cd concentration (**Figure 3D**), and it was closely related to CAT and POD activities (**Figure 6B**). The increase in the content of MDA could lead to the accumulation of a large amount of ROS, which stimulates the generation of unsaturated fatty acids in the lipid bilayer membrane, leading to lipid membrane peroxidation.

### 4.3 Phyllosphere bacterial diversity and key Cd tolerance-related DAMs in *T. tortuosa*

The phyllosphere of plants contacts with external environment (Liu et al., 2020). There are many microorganisms in the phyllosphere, which impact plant physiological activities and growth (Brandl et al., 2023). Some phyllosphere bacteria affect plant growth by impacting plant pathogens and nitrogen fixation and reducing the damage of pollutants (Qian et al., 2023). Qian et al. (2023) reported that Proteobacteria had the highest relative abundance in rice phyllosphere under Cd stress, followed by Firmicutes and Actinobacteriota. Yang et al. (2022) reported that Bacteroidetes, Proteobacteria, and Cyanobacteria were the dominant species in duckweed phyllosphere. Similarly, in this study, Cyanobacteria and Proteobacteria were the dominant species in the phyllosphere under Cd stress (10 mg·L^-1^) (**Figure 4B**). Cyanobacteria has various surface layers, and each layer has specific molecular groups that provide massive sites for binding to heavy metals. Therefore, Cyanobacteria has a strong heavy metal adsorption capacity (Kalita and Baruah, 2023).

Consistent with the results of Qian et al. (2023), in this study, the relative abundances of phyllosphere bacteria were negatively correlated with the plant Cd concentration under Cd exposure, and plant Cd concentration was negatively correlated with the relative abundances of Acidobacteriota, Actinobacteriota, Armatimonadota, Bdellovibrionota, Chloroflexi, Firmicutes, Planctomycetota, and Verrucomicrobiot (*p* < 0.05) (**Table 1**). This indicates that Cd stress inhibits the phyllosphere bacterial activity of *T. tortuosa*. In addition, in this study, the relative abundances of phyllosphere bacteria (Actinobacteriota, Bdellovibrionota, Chloroflexi, Planctomycetota, and Verrucomicrobiota) were positively correlated with the relative abundances of DAMs (Neomycin, N-Acetyl-L-Glutamic acid, Fusaric acid, 5-Hydroxy-L-tryptophan, and Serotonin) (**Table 2**; **Figure 6B**). Among the DAMs, fusaric acid is a toxin generated by *Fusarium* species. It can quickly lead to temporary membrane hyperpolarization by inducing necrotic leaf spots, leaf, stem, and petiole wilting, and suppressing root and root hair growth (Bouizgarne et al., 2006; Dresler et al., 2022). Therefore, Cd stress has a great negative impact on *T. tortuosa* and its phyllosphere bacteria. Some studies have also shown that the content of allantoin in bryophytes such as *Brachythecium rutabulum*, *Hypnum cupressiforme*, and *Tortula muralis* significantly increased with the increase of heavy metal concentration. This may be another adaptive mechanism of bryophytes in addition to secretion of fusaric acids (Dresler et al., 2022). In this study, production of neomycin, L-tryptophan, N-Acetyl-L-Glutamic acid and its derivative 5-Hydroxy-L-tryptophan is also a mechanism of *T. tortuosa* to resist exogenous Cd stress (Chaffai et al., 2006). The bacterial function prediction results showed that the functions of dominant bacteria were mainly amino acid transport and metabolism (**Supplementary Figure S3**). This indicates that amino acid is the core metabolite in *T. tortuosa* in the resistance to exogenous Cd stress. In summary, Cd stress could decrease the relative abundances of phyllosphere bacteria Cyanobacteria, Proteobacteria, and Acidobacteriota, and the relative abundances of dominant bacteria Chloroflexi, Verrucomicrobiota, and Actinobacteriota were negatively correlated with the Cd concentration in *T. tortuosa*. The toxic effect of exogenous Cd on *T. tortuosa* was manifested in an increase in fusaric acid.

## 5 Conclusion

*Tortella tortuosa* had a strong Cd absorption capacity (maximum: 5.0135 mg·kg^-1^) and can therefore be used for phytoremediation of heavy metal pollution. The physiological responses of *T. tortuosa* to Cd stress included decreases in *Chl a* content, *Chl b* content, CAT activity, and POD activity. Another responses included changes in phyllosphere bacterial community composition, decreases in the relative abundances of dominant phyllosphere bacteria (Proteobacteria, Acidobacteriota), and secretion of amino acid L-Tryptophan and its derivatives N-Acetyl-L-Glutamic acid and 5-Hydroxy-L-tryptophan. In future studies, the absorption capacity for different heavy metals of different bryophytes as well as the molecular mechanisms of bryophytes responding to heavy metal stresses will be explored. This study provides a reference for Cd pollution remediation.

## Data Availability

The data presented in the study are deposited in the NCBI repository, accession number PRJNA1191150 (http://www.ncbi.nlm.nih.gov/bioproject/1191150).
