# Supplementary material for: Phyllosphere bacterial community and metabolomic analysis revealed the mechanism of Cd tolerance in the bryophyte Tortella tortuosa (Hedw.) Limpr
Source: Front Plant Sci. 2024 Nov 28;15:1466659. doi: 10.3389/fpls.2024.1466659 (PMC11635300; doi:10.3389/fpls.2024.1466659)
Supplement: Supplementary file 1 [file DataSheet1.docx]

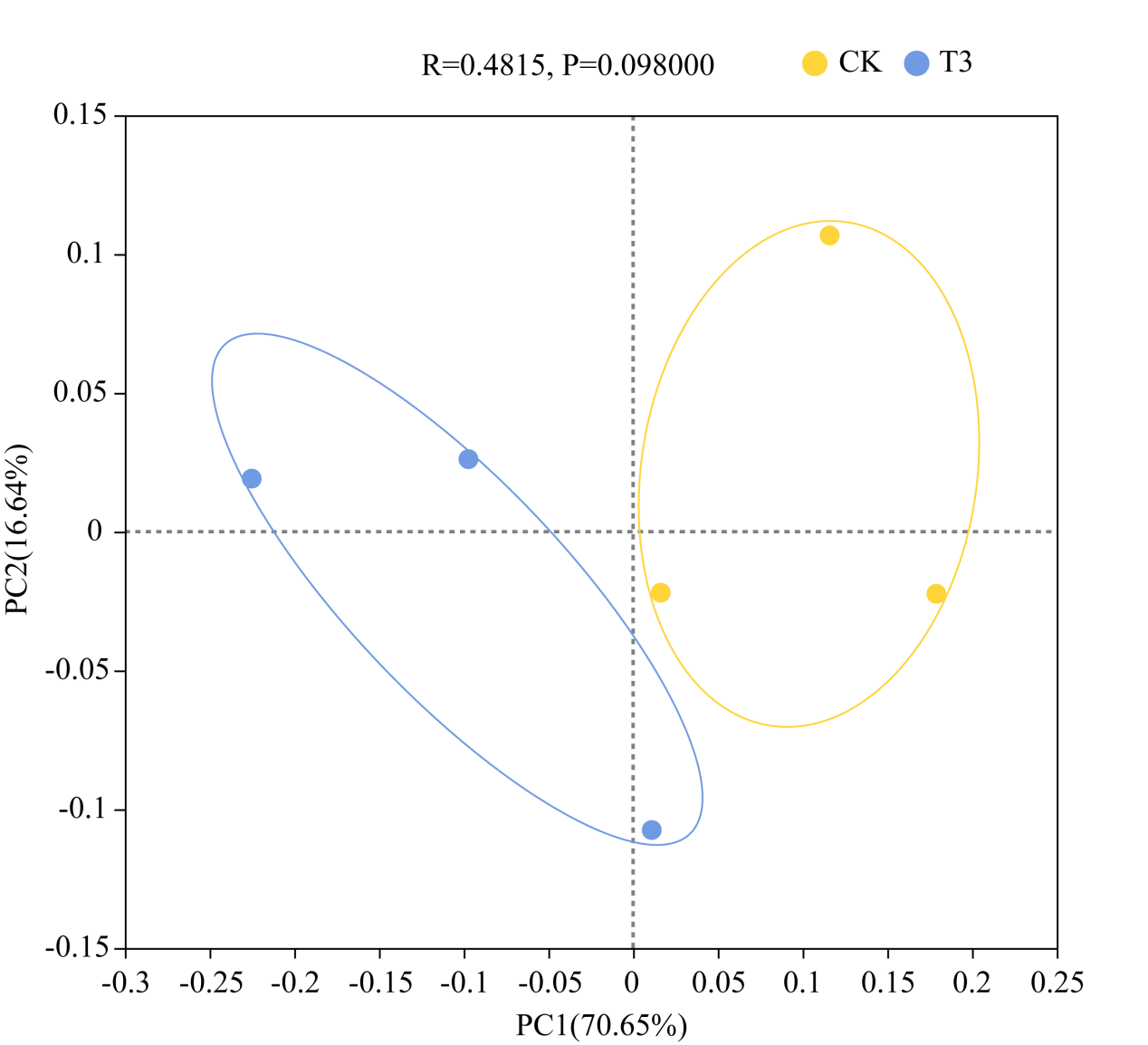


**Fig. S1** Analysis results of phyllospheric bacterial β-diversity of *T. tortuosa* (species level)


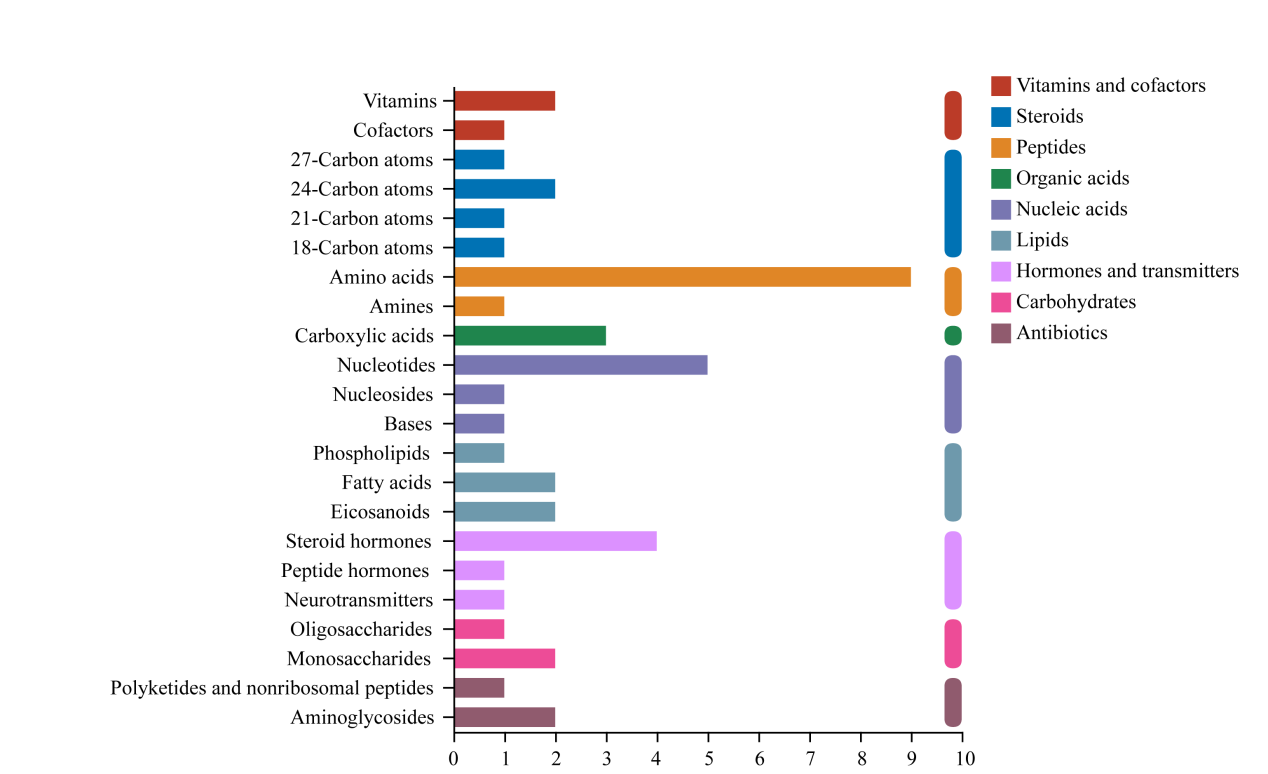


**Fig. S2** Classification and statistics of differentially abundant metabolites (DAMs)


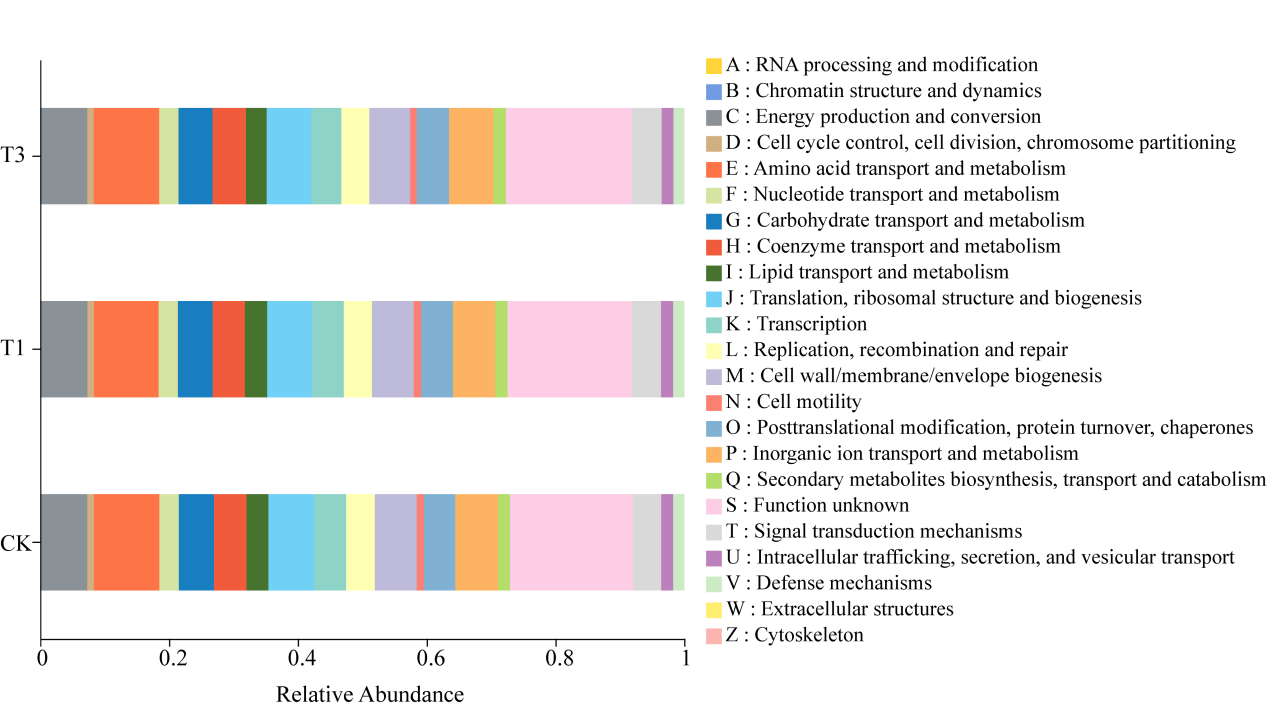


**Fig. S3** Prediction of phyllosphere bacterial diversity of *T. tortuosa* based on PICRUSt2
